# Treatment of idiopathic granulomatous mastitis and factors related with disease recurrence

**DOI:** 10.3906/sag-2003-93

**Published:** 2020-08-26

**Authors:** Emre TEKGÖZ, Seda ÇOLAK, Muhammet ÇINAR, Sedat YILMAZ

**Affiliations:** 1 Department of Internal Medicine, Division of Rheumatology, Gülhane Faculty of Medicine, University of Health Sciences Turkey, Ankara Turkey

**Keywords:** Azathiopurine, idiopathic granulomatous mastitis, methotrexate, treatment

## Abstract

**Background/aim:**

Idiopathic granulomatous mastitis is a rare, benign inflammatory disease of breast. There is no general agreement on the appropriate treatment choice. The aim of the study was to investigate the immunosuppressive administer for idiopathic granulomatous mastitis and risk factors related with disease recurrence.

**Materials and methods:**

The data of 53 patients with idiopathic granulomatous mastitis were evaluated for this cross-sectional retrospective study. Demographic features and clinical characteristics and course of the patients were obtained from file records.

**Results:**

The mean age of the patients was 37.2 ± 6.6 years. Fifty-one of 53 patients received immunosuppressive treatment with or without surgery. Forty-seven (88.6%) of the patients received only immunosuppressive treatment without surgery, while 4 (7.54%) patients received immunosuppressive treatment after surgery. Forty-one (77.3%) of 47 patients who had no surgical resection received methotrexate as immunosuppressive treatment. The other 6 (11.3%) patients received azathioprine or corticosteroid treatment. Complete or partial remission was observed in 50 (98%) of 51 patients who received immunosuppressive treatment, while only 1 (2%) patient did not reach remission. No factors were found related with recurrence of disease.

**Conclusion:**

Methotrexate seems to be efficient in the treatment of idiopathic granulomatous mastitis and provides drug-free remission.

## 1. Introduction

Idiopathic granulomatous mastitis (IGM) is a rare and benign chronic inflammatory disease of breast with an unknown etiology [1]. Due to clinical presentations (mass, erythema, swelling, fistula formation) and radiological findings (abscess formation, enlarged lymph nodes, hypoechoic lesions, calcifications, and asymmetric increased density), it may be confused with carcinomas or infections [2]. Histopathological examination is warranted to show noncaseating granulomatous lobulitis. Diseases which may cause chronic granulomatous lobulitis such as tuberculosis, granulomatous with polyangiitis, and sarcoidosis should be excluded before the patients were diagnosed with IGM. In the literature, there is limited data about treatment approach of IGM [3,4]. Surgery and/or medical therapy including antibiotics and immunosuppressive agents are treatment choices, as well as there is no established treatment algorithm for IGM [5,6]. This study aims to investigate the treatment choices and follow up data of the patients with IGM. 

## 2. Materials and methods

### 2.1. Study design, sample, and setting

This retrospective study was conducted in a tertiary rheumatology outpatient clinic. It was approved by the Ethical Review Board of Gülhane Training and Research Hospital.

A total of 53 patients who had noncaseating granulomas and were diagnosed with IGM after physical examination and histopathologic evaluation of the breast biopsy samples were included in the study. The patients were followed up in our outpatient clinic between February 2011 and September 2018. The patients diagnosed with other granulomatous diseases were excluded. All of the patients included in the study were female due to the higher frequency of disease among female patients. Informed consent form was obtained from each participant.

### 2.2. Data collection 

Demographic and clinical data were obtained retrospectively from patient files. The treatment options applied (antibiotics, steroids, immunosuppressive drugs, or surgical intervention), the duration, course, and outcomes of treatment for each patient were investigated. 

Treatment efficiency was evaluated with clinical improvement and partial or complete remission. In the first month visit, presence of any improvement in symptoms, physical findings, and acute phase reactants were accepted as clinical improvement. The presence of at least 50% improvement in clinical and radiological findings at 3rd or 6th month visits was accepted as partial remission. Besides, complete recovery in clinical and radiological findings at 6th month visit was accepted as complete remission.

### 2.3. Data analysis

For statistical analyses, IBM SPSS 24 software for Windows (SPSS Inc., Chicago, Illinois) was used. Descriptive statistics were presented as mean ± standard deviation and median (minimum–maximum) values for measured variables, and frequency and percentage (%) for categorical data. Categorical variables of the patients with or without recurrence were compared by using Pearson’s chi-squared test and Fisher’s exact test as appropriate. A value of P < 0.05 was accepted as statistically significant.

## 3. Results 

All patients included in the study were female in childbearing age. The mean age was 37.2 ± 6.6 years. The median time to be diagnosed with IGM was 4.37 months (range: 0.7–144 months) and median time of follow-up was 13.83 months (range: 1.61–100.83 months). Fifty (94.3%) patients were diagnosed within the first 2 years of breastfeeding. At the time of diagnosis, three (5.6%) patients were pursuing breastfeeding and one (1.8%) patient was pregnant. The most common symptom in the admission was palpable mass in the breast in all of the patients. Other symptoms were changes on the skin of breast in 38 (71.7%), fistula and purulent discharge in 30 (56.5%), and ulceration on the skin of breast in 11 (20.8%) patients. Among 25 (47.2%) patients, the left breasts were influenced, whereas among 23 (43.4%) patients the right breasts and in 5 (9.4%) patients both of them were affected. Axillary reactive lymphadenopathies were detected in 21 (39.6%) of the patients. Detailed demographic and clinical data of the patients were presented in Table 1. 

**Table 1:** Demographic and clinical characteristics of the patients (n = 53).

Patients’ characteristics	Value
Age, years†	37.2 ± 6.6
Disease duration, months‡	4.37 (0.7–144)
Follow-up duration, months‡	13.83 (1.61–100.83)
Body mass index, kg/m^2^, n (%) ≥ 30 25-30 <25	12 (22.6) 29 (54.8) 12 (22.6)
Smoker, n (%)	20 (37.7)
Number of pregnancies 0 1 >1	3 (5.7) 13 (24.5) 37 (69.8)
Patients using contraception, n(%) Oral contraceptive pills Intrauterine device	21 (39.6) 12 (22.6) 9 (17.0)
Main complaint, n (%) Palpable mass Skin changes (erythema, nipple inversion, induration) Fistula formation and purulent drainage Ulceration Axillary lymphadenopathy	53 (100) 38 (71.7) 30 (56.5) 11 (20.8) 21 (39.6)
Affected breast, n (%) Unilateral- left Unilateral- right Bilateral	25 (47.2) 23 (43.4) 5 (9.4)

† mean ± standard deviation, ‡median (minimum–maximum), other values are presented as percentage

Initial treatment options applied to the patients were presented in Table 2. Forty-seven (88.7%) patients received only immunosuppressive treatment, 4 (7.5%) patients received immunosuppressive treatment following surgical intervention, and one (1.9%) patient underwent surgical resection without any immunosuppressive treatment. Only one (1.9%) patient denied receiving treatment. 

**Table 2:** Initial treatment approaches of the patients.

	n	%
Surgical intervention	5	9.4
Only surgery	1	1.9
Immunosuppressive treatment after unsuccessful surgery	2	3.8
Immunosuppressive treatment with surgery	2	3.8
Immunosuppressive drugs	47	88.7
Only corticosteroids	3	5.7
Methotrexate and corticosteroids	41	77.4
Azathioprine and corticosteroids	3	5.7
No treatment	1	1.9

Among 47 patients who received only immunosuppressive drugs for initial treatment, 41 (77.4%) received methotrexate (MTX) and corticosteroids (CS), 3 (5.7%) received azathioprine (AZA) and CS, and 3 (5.7%) received only CS. Surgical intervention was performed as initial treatment for 5 (9.4%) patients. Median doses of MTX, AZA, and CS were 15 mg/week, 125 mg/day, and 40 mg/day, and median durations of treatment were 9.1, 5.61, and 9.63 months, respectively.

Clinical improvement was observed in the first month of the treatment in all of 41 patients who received MTX and CS for the initial treatment. Among the 41 patients who received MTX for initial treatment, 36 (87.8%) continued receiving MTX throughout the treatment process. Thirty-three (80.5%) of those 36 patients were found to be in sustained remission in the follow-up visits. Median time of achieving remission was 13.04 months (range: 1–62 months). After remission, 3 (9.1%) patients were followed up with only MTX, 16 (48.5%) patients with MTX and CS, and 14 (42.4%) patients were followed drug-free. On the other hand; 3 (7.3%) patients were accepted to be in partial remission according to the improvement of clinical symptoms, in spite of short treatment durations (shorter than 6 months). During follow-up visits, recurrent disease was detected in both clinic and ultrasonographic evaluations of 5 (12.2%) patients who were receiving MTX. Recurrence was determined in 4 (9.8%) patients at 3rd month of the treatment process. Clinical and radiological recurrences were detected in all these 5 patients at 6th month of treatment. One (2.44%) of them treated with higher doses of MTX and CS, and treatments of other 4 (9.76%) patients were switched to AZA. The mean time of switching from MTX to AZA was 8.72 ± 2.3 months. In two (4.88%) patients whose treatments were switched to AZA due to the presence of recurrence, remission was observed in 10th and 26th months of the treatment process. One patient had gastrointestinal symptoms with MTX, and the treatment was switched to AZA. This patient also had remission with AZA treatment. 

Two of three patients (66.7%) who received AZA and CS for initial treatment had clinical improvement in the first month of therapy and no recurrence was seen during follow-up visits. These two patients achieved both clinical and radiological remissions in 6th and 9th months of the treatment duration, respectively. One patient had progression of clinical symptoms. Due to concomitant Sjogren’s syndrome and thrombocytopenia in this patient, cyclosporine-A was applied. In total of 7 patients who received AZA, complete remission was observed in five (71.4%) and partial remission was observed in 2 (28.6%) patients. Median time for remission was 9.33 months (range: 6–26 months). Treatment of AZA could be withdrawn in none of the patients. 

Three (5.7%) patients received CS for initial treatment. All of these patients had clinical improvement in the 1st month of the treatment. All of the patients’ clinical and radiological remissions were observed in a median time of 9.63 months (range: 1–13 months). Among these 3 patients, CS treatment was stopped for one and tapered in the follow-up period of the other two patients. 

After surgical invention, 4 patients had received MTX. Two of the patients received MTX after operation and the other two had recurrence after surgery and MTX was started after recurrence. One patient used no immunosuppressive treatment after surgery. Relapse after surgery was observed in all of 3 patients who had no immunosuppressive treatment. The longest remission time after surgical intervention was 32.75 months without immunosuppressive treatment. Although one patient had no treatment (either medical or surgery), clinical and radiological remission were achieved after 12 months follow-up. After 64 months follow-up, a sustained remission was observed in this patient.

Fifty-one patients had immunosuppressive treatment for IGM. Complete remission was seen in 45 (88.2%), partial remission was seen in 5 (9.8%), and no remission was seen in 1 (2%) of the patients. Median time for remission was 11.99 months (range: 1–62 months). Clinical and radiological responses of the patients were shown in Table 3. 

**Table 3:** Responses of the patients during follow-up.

	Initial	1st monthfollow-up	3rd monthfollow-up	6th monthfollow-up
Palpable mass, n (%)	53 (100)	1 (1.9)	5 (9.4)	19 (35.8)
Skin changes, n (%)	38 (71.7)	1 (1.9)	5 (9.4)	4 (7.5)
Ulceration, n (%)	11 (20.8)	0	0	0
Fistula formation and purulent drainage, n (%)	30 (56.5)	1 (1.9)	1 (1.9)	0
Diameter of mass, mean ± SD	2.5 ± 1.0		1.2 ± 0.5	0.5 ± 0.3

Factors related with disease recurrence were investigated. No statistically significant difference in terms of smoking, parity, body mass index, and use of oral contraceptive pills was detected between recurrent IGM and nonrecurrent IGM groups (P values were 0.748, 1.0, 0.711, and 0.743, respectively). 

## 4. Discussion

IGM is known to be a rare and benign inflammatory disease of breast tissue, although the etiology remains to be unclear [7]. Despite the increasing frequency of patients diagnosed with IGM in recent years, there is still limited data about the clinical course and management of the disease. 

IGM primarily affects young women in the lactation period [8]. Lactational secretions may cause a local granulomatous reaction [9]. Also, pituitary adenoma (prolactinomas) and some drugs (e.g., antipsychotics) may increase intraductal secretion. Although exact etiological factors are not clarified yet, obesity, smoking, oral contraception pills, ethnicity, and infectious agents are suspected as etiological factors [10]. 

IGM may cause ulcers or abscess and mimic breast cancer. Breast biopsy is needed to clarify histopathological examination in order to exclude cancers or infections. Biopsy findings typically include noncaseous granulomatous lesions with leukocytosis, epithelioid cells, and macrophages, located in the center of breast lobule [11]. Although IGM is accompanied with noncaseous necrosis, acid-fast staining technique and tissue culture should be performed especially in tuberculosis-endemic areas [12]. In the present study, tissue biopsy and culture are performed for all patients to exclude diseases other than IGM. 

In the current study, all of the patients had unilateral palpable mass in the breast; skin changes of the breast and fistula formation were the other two common symptoms. These findings were similar to those in the literature [13,14]. All patients underwent mammography, breast ultrasonography (USG), and magnetic resonance imaging (MRI) as radiological evaluation at the time of diagnosis. USG and MRI findings provide important information about IGM [15]. Mammography may demonstrate masses which lead to asymmetrical increase in the density of the breast and axillary lymphadenopathies. Ultrasonography may show multiple confluent, irregular, hypoechoic masses. Frequently, fistula or abscess formation may be observed in USG or MRI. Irregular heterogeneous lesions and parenchymal distortion with a hyperintense signal on T2-weighted and a variable signal on T1-weighted images are MRI findings of IGM [15]. However, none of these imaging modalities are superior to biopsy in the diagnosis of IGM [7]. Radiological evaluation is important in diagnosis as well as in follow-up visits. In this study, all patients were evaluated with USG initially, at 6th and 12th months. Both clinical and radiological regression was evaluated during follow-up of the patients. 

Although there are multiple approaches regarding the management of IGM in the literature, there is no clear consensus for treatment. Treatment options include antibiotherapy, surgical excision, and systemic immunosuppressive agents such as CS, MTX, and AZA. Although IGM is a benign, self-limiting disease, disease recurrence is reported to be approximately 50% among the patients who had not received treatment [16]. In our study, one patient accepted neither surgical nor immunosuppressive treatment. In this patient, remission was observed after 12 months. The patient was followed closely because of the high relapse rate in the literature and no recurrence observed during the follow up period of 64 months. Many centers prefer empirical antibiotic therapy at the beginning or concurrent with surgery in case of the abscess formation and open draining sinuses. The relationship between IGM and *Corynebacterium* infection was presented in the literature and antibiotic therapy seems to be effective in these cases [9,17]. In our study, 30 (56.6%) patients with fistula formation and purulent drainage received antibiotic therapies, but no improvement was detected. Tests for *Corynebacterium* in these patients were also negative. On the other hand, most common complications of unsuccessful surgical operations are relapse, fistula formation, and secondary infection [18]. In our study, 5 (9.4%) patients underwent surgical intervention and, 3 of them received no immunosuppressive treatment after surgery. Disease recurrence was reported in these 3 patients. 

The most commonly chosen systemic immunosuppressive drugs are CS, MTX, and AZA in the treatment of IGM [19]. Studies in the literature have shown that CS treatment may be effective in reducing the size of the mass and improving abscess formation in patients with IGM [20–23]. However, many side effects can be observed with long-term and high-dose CS treatment. Additionally, studies in the literature reported that approximately 50% recurrence rate is possible while decreasing the dosage of CS [24]. In our clinic, 40–60 mg/day prednisolone therapy for initial treatment is usually preferred. After evaluating the clinical response within 2–4 weeks, MTX is added to CS treatment and CS dose is reduced rapidly. After remission, immunosuppressive treatment can be stopped in 16–24 months. This approach allows minimizing side effects associated with CS treatment.

In the literature there are studies showing the effectiveness of MTX in reducing disease recurrence, decreasing CS dose as well as providing disease remission [5,19,25,26]. Regarding a review which analyzed treatment of IGM patients retrospectively, implementation of MTX is effective in suppressing inflammation, preventing complications and reducing the side effects of CS [3]. As shown in the current study, MTX seems to be efficient in reducing disease recurrence and providing drug-free remission. Furthermore, to the best of our knowledge, our cohort consisting of 51 patients receiving systemic immunosuppressive therapy is the largest sample in the literature. Similarly, Konan et al. showed the effectiveness of AZA in the treatment of IGM [27]. In our study, among the 7 patients who were treated with AZA due to pregnancy or MTX-resistant disease, remission was observed in 5 patients.

In our study, 5 patients were considered to have partial remission. Actually, there is no exact definition about partial remission in IGM patients. In our clinic practice, patients who had improvement in clinical and radiological sign and symptoms by 50% and above were accepted as in partial remission, according to the evaluation in 3rd and 6th months. However, patients with partial response were indeed the patients with short follow-up period. The frequency of complete response may increase with the extension of follow-up duration. Besides, it may be speculated that expecting higher response rates in longer follow-up duration will not be surprising. 

Previous studies have indicated that breastfeeding may be the main factor responsible for IGM, especially in reproductive age [9,19]. In the current study, 50 (94.3%) of the patients had a history of one or more pregnancies and all of these patients had the diagnosis of IGM in the first 2 years following breastfeeding onset. Twelve of 50 patients who have at least one pregnancy history had disease recurrence. It is found that there is no difference in parity and breastfeeding between the groups in relation to recurrence. According to a study performed by Uysal et al., breastfeeding and pregnancy were suggested to be risk factors for recurrence of IGM [13]. 

Oral contraceptive pills are known to be one of the risk factors for IGM, in relation with increased secretion from the breast. Hanna et al. showed that patients with chronic mastitis had higher rates of contraception utilization than the control group [14]. In our study, 21 (39.6%) patients were using oral contraceptive pills or hormonal intrauterine device for contraception. The frequency of contraceptive pills was found to be higher than other contraception methods, similar to the literature [14]. Among 21 patients who were using oral contraceptive pills or intrauterine hormonal devices, 4 had recurrent disease. There was no statistically significant association between recurrence of IGM and contraception utilization, consistent with previous research [13,28]. 

In recent studies, smoking was reported as an effective factor in the etiology of IGM [4,10,29]. Although there were 20 patients who were smokers, the number of patients with recurrent disease was 5. No statistically significant difference was found in terms of smoking between recurrent and nonrecurrent groups. Likewise, Yilmaz et al. showed that smoking was not significantly different between groups with or without recurrence [28]. On the other hand, Uysal et al. and Co et al. investigated the role of smoking in the recurrence of disease and according to the findings of both studies, smoking is a prognostic factor for IGM recurrence [13,28,30]. 

There were 12 patients with BMI above 30. Two of them had recurrence of disease and there was no statistically significant difference in terms of obesity between recurrent and nonrecurrent groups. To our knowledge, there is no data in the literature investigating the relationship between obesity and IGM. Actually, there is limited data in the literature investigating the factors related with recurrence of IGM.

In our clinic, we prefer MTX treatment as a CS tapering agent in initial treatment. AZA is preferred for the patients for whom MTX is not appropriate. We obtained complete remission in 45 (88.2%) of 51 patients who were receiving immunosuppressive treatment. According to the current study, MTX seems to be an effective choice for providing rapid tapering of the steroid dose, and drug-free remission.

Systemic immunosuppressive drugs are important in the management of patients with IGM. Addition of MTX to CS therapy can be efficient in reducing CS dose, controlling the inflammatory process, providing remission, and preventing further complications. Surgical treatment may be conceivable for patients with recurrent or drug-resistant disease. The main limitation of our study is its retrospective design. Further studies with larger sample and prospective design are needed to confirm the efficacy of systemic immunosuppressive therapies in the treatment of IGM. 

## References

[ref1] (1972). Granulomatous mastitis: a lesion clinically simulating carcinoma. American Journal of Clinical Pathology.

[ref2] (2012). Differential diagnosis in idiopathic granulomatous mastitis and tuberculous mastitis. Journal of Breast Cancer.

[ref3] (2011). Methotrexate in the management of idiopathic granulomatous mastitis: review of 108 published cases and report of four cases. Breast Journal.

[ref4] (2014). Idiopathic granulomatous mastitis : comparison of wide local excision with or without corticosteroid therapy. Breast Care.

[ref5] (2003). Methotrexate in the management of granulomatous mastitis. ANZ Journal of Surgery.

[ref6] (2012). Management of granulomatous mastitis : a series of 14 patients. Gynecological Endocrinology.

[ref7] (2013). Management of idiopathic granulomatous mastitis diagnosed by core biopsy: a retrospective multicenter study. The Breast Journal.

[ref8] (2000). Granulomatous mastitis in pregnancy. Obstetrics and Gynecology.

[ref9] (2003). A clinicopathological review of 34 cases of inflammatory breast disease showing an association between corynebacteria infection and granulomatous mastitis. Pathology.

[ref10] (2008). Idiopathic granulomatous mastitis : a 25-year experience. Journal of the American College of Surgeons.

[ref11] (2017). Idiopathic granulomatous mastitis: dilemmas in diagnosis and treatment. Electronic Physician.

[ref12] (2011). Recurrent granulomatous mastitis mimicking inflammatory breast cancer. BMJ Case Reports.

[ref13] (2018). Factors related to recurrence of idiopathic granulomatous mastitis: what do we learn from a multicentre study. ANZ Journal of Surgery.

[ref14] (2013). Risk factors for chronic mastitis in Morocco and Egypt. International Journal of Inflammation.

[ref15] (2013). Chronic granulomatous mastitis : imaging , pathology and management. European Journal of Radiology.

[ref16] (2005). The role of conservative treatment in idiopathic granulomatous mastitis. The Breast Journal.

[ref17] (2016). Cystic neutrophilic granulomatous mastitis: association with gram-positive bacilli and corynebacterium. American Journal of Clinical Pathology.

[ref18] (2006). Granulomatous mastitis including breast tuberculosis and idiopathic lobular granulomatous mastitis. Canadian Journal of Surgery.

[ref19] (2015). Treatment for and clinical characteristics of granulomatous mastitis. Obstetrics and Gynecology.

[ref20] (2005). Idiopathic granulomatous mastitis: a case successfully treated with a minimum dose of a steroid. Chang Gung Medical Journal.

[ref21] (2007). Three patients with granulomatous mastitis showing good response to oral prednisolone. The Ceylon Medical Journal.

[ref22] (2009). Vacuum-assisted biopsy and steroid therapy for granulomatous lobular mastitis: report of three cases. Surgery Today.

[ref23] (2009). Granulomatous lobular mastitis: imaging, diagnosis, and treatment. American Journal of Roentgenology.

[ref24] (2003). Chronic granulomatous mastitis: diagnostic and therapeutic considerations. World Journal of Surgery.

[ref25] (2011). Is methotrexate an acceptable treatment in the management of idiopathic granulomatous mastitis?. Archives of Gynecology and Obstetrics.

[ref26] (2004). Rheumatologists and breasts: immunosuppressive therapy for granulomatous mastitis. Rheumatology.

[ref27] (2012). Combined long-term steroid and immunosuppressive treatment regimen in granulomatous mastitis. Breast Care.

[ref28] (2018). Scoring idiopathic granulomatous mastitis : an effective system for predicting recurrence. European Journal of Breast Health.

[ref29] (2007). Idiopathic granulomatous mastitis: a heterogeneous disease with variable clinical presentation. World Journal of Surgery.

[ref30] (2018). Idiopathic granulomatous mastitis: a 10-year study from a multicentre clinical database. Pathology.

